# Sex differences in behavioural and anatomical estimates of visual acuity in the green swordtail, *Xiphophorus helleri*

**DOI:** 10.1242/jeb.243420

**Published:** 2021-12-17

**Authors:** Eleanor M. Caves, Fanny de Busserolles, Laura A. Kelley

**Affiliations:** 1 University of Exeter, Centre for Ecology and Conservation, Penryn TR10 9FE, UK; 2 Department of Ecology, Evolution, and Marine Biology, University of California Santa Barbara, Santa Barbara, CA 93106, USA; 3 The University of Queensland, Queensland Brain Institute, Brisbane 4072, Australia

**Keywords:** Visual signal, Retinal topography, Spatial resolution, Sensory ecology, Optomotor

## Abstract

Among fishes in the family Poeciliidae, signals such as colour patterns, ornaments and courtship displays play important roles in mate choice and male–male competition. Despite this, visual capabilities in poeciliids are understudied, in particular, visual acuity, the ability to resolve detail. We used three methods to quantify visual acuity in male and female green swordtails (*Xiphophorus helleri*), a species in which body size and the length of the male's extended caudal fin (‘sword’) serve as assessment signals during mate choice and agonistic encounters. Topographic distribution of retinal ganglion cells (RGCs) was similar in all individuals and was characterized by areas of high cell densities located centro-temporally and nasally, as well as a weak horizontal streak. Based on the peak density of RGCs in the centro-temporal area, anatomical acuity was estimated to be approximately 3 cycles per degree (cpd) in both sexes. However, a behavioural optomotor assay found significantly lower mean acuity in males (0.8 cpd) than females (3.0 cpd), which was not explained by differences in eye size between males and females. An additional behavioural assay, in which we trained individuals to discriminate striped gratings from grey stimuli of the same mean luminance, also showed lower acuity in males (1–2 cpd) than females (2–3 cpd). Thus, although retinal anatomy predicts identical acuity in males and females, two behavioural assays found higher acuity in females than males, a sexual dimorphism that is rare outside of invertebrates. Overall, our results have implications for understanding how poeciliids perceive visual signals during mate choice and agonistic encounters.

## Abstract (precis)

**Summary:** Anatomical and behavioural quantification of visual acuity (spatial resolving power) in green swordtails indicates that acuity was anatomically identical in both sexes, but behaviourally higher in females, with implications for signalling.

## INTRODUCTION

Many animals use signals to communicate in interactions such as mate choice and aggression, and one important selective pressure on the form of those signals is the perceptual system of the receiver. In the case of visual signals, understanding how a signal is perceived requires knowledge of the receiver's visual capabilities, including visual acuity, the ability to perceive detail. Visual acuity (which is sometimes referred to as spatial resolving power) is an ecologically and behaviourally important visual parameter, as it determines what can and cannot be resolved in a given scene (Cronin et al., 2014). Thus, quantifying visual acuity can provide insight into what visual information a species can extract from its environment, and thus how it might place selection pressure on visual signals (Caves et al., 2018). Broadly, acuity can be estimated anatomically (by measuring the density and spacing of cells in the retina) or behaviourally (using trained or innate responses). Although studies directly comparing behavioural and anatomical acuity are rare, in those studies that do exist, there is inconsistency in whether the methods yield comparable estimates (e.g. Goller et al., 2019; Harman et al., 1986; Temple et al., 2013) or different estimates (e.g. Champ et al., 2014; Parker et al., 2017). Comparing anatomical and behavioural acuity can provide insights into how retinal anatomy as well as post-retinal processing can influence a species' ability to discriminate detail.

Visual acuity is highly variable across species, varying by at least four orders of magnitude across animals with image-forming eyes (Caves et al., 2018). Acuity is reported in units called cycles per degree (cpd), which is the number of pairs of black and white stripes a viewer can resolve in a single degree of visual angle (higher values in cpd indicate sharper vision). Some of the highest acuities are found in birds of prey (wedge-tailed eagle, *Aquila audax*: 140 cpd; Reymond, 1985) and in humans (approximately 60 cpd; e.g. Campbell and Gubisch, 1967; Curcio and Allen, 1990), while the lowest are found in small compound eyes such as those of *Drosophila melanogaster* (0.01 cpd; Gonzalez-Bellido et al., 2011). Among ray-finned fishes, published measures of acuity range from approximately 1 cpd in the Japanese rice fish, *Oryzias latipes* (Carvalho et al., 2002), to approximately 30 cpd in species such as the collared large-eye bream, *Gymnocranius audleyi* (Collin and Pettigrew, 1989), and the sailfish *Istiophorus albicans* (Tamura and Wisby, 1963). Aside from eye type (e.g. camera eye or compound eye), the primary factor underlying variation in acuity across species is variation in eye size, with higher acuity typically found in larger eyes (Caves et al., 2018). In ray-finned fish, variation in eye size explains approximately 55% of variation in acuity (Caves et al., 2017). Various ecological parameters are also important correlates of acuity in fish. In particular, species that live in spatially complex habitats such as coral reefs and kelp forests have higher acuity relative to their body size than those that live in horizon-dominated or featureless habitats (Caves et al., 2017; Dobberfuhl et al., 2005).

Anatomically, limits on acuity can be imposed by the optical properties of the lens and the spacing of the retinal neurons. In fish, however, anatomical estimates of acuity mostly depend on the retinal morphology, because fish have very accommodating lenses (Douglas and Hawryshyn, 1990). In terms of retinal morphology, the retinal ganglion cells (RGCs), which are the final stage of retinal processing and are the only retinal cells that connect to the brain, represent the upper limit of spatial resolving power (Collin and Pettigrew, 1989). To measure RGC density, one can examine retinal topography using the retinal whole-mount technique (Stone, 1981; Ullmann et al., 2012), which allows the analysis of the entire retina and the identification of areas of high cell density or retinal specializations. These specializations, which provide higher acuity in a specific part of the visual field, are usually classified into two main types, ‘areas’ and ‘streaks’, where the increase in cell density is concentric or elongated across the retinal meridian, respectively. Variability in the type of specialization and its localization often correlates with the ecology and behaviour of a particular species and has been shown in fish to vary with habitat type, feeding ecology, predator or conspecific detection, and life stage (Collin and Pettigrew, 1988a,b; de Busserolles et al., 2014, 2021; Luehrmann et al., 2020; Shand et al., 2000; Stieb et al., 2019; Tettamanti et al., 2019).

Behaviourally estimating acuity can be accomplished by assessing either the innate or trained responses of an animal. For example, the optomotor response, a taxonomically widespread innate response to wide-field visual stimulation, can be elicited by placing an animal in a rotating drum surrounded by a grating of vertical black and white stripes (see Caves et al., 2020). The striped grating is rotated, and if the animal can resolve the stripes, it reflexively turns or moves its body, head or eyes to track the rotation (McCann and MacGinitie, 1965). If an animal cannot resolve the stripes – i.e. they are below its acuity limit – the optomotor response is absent. Another way to behaviourally measure acuity involves conditioning an animal to associate either a grating of black and white stripes or a uniform grey stimulus of the same mean luminance with a food reward. The ability of the animal to discriminate the stimuli is then tested with grating stimuli of varying spatial frequency (e.g. Baerends et al., 1960; Brünner, 1934; Champ et al., 2014; Parker et al., 2017; Penzlin and Stubbe, 1977). The spatial frequency where the animal can no longer reliably distinguish the two stimuli is its acuity limit.

Each of these methods has advantages and disadvantages. The primary disadvantage of anatomical measures is that they cannot account for diffraction and other optical imperfections (e.g. Browman et al., 1990; Haug et al., 2010; Lee and O'Brien, 2011), spatial and temporal summation, or other forms of higher-order neural processing of visual signals, all of which may limit the resolution of an eye. Moreover, errors in cell differentiation during the retinal mapping analysis, resulting in the inclusion of ‘displaced’ amacrine cells or the omission of small ganglion cells, could over/underestimate cell densities and therefore visual acuity measures (Collin and Pettigrew, 1989; Pettigrew and Manger, 2008; Pettigrew et al., 1988). Behavioural methods, however, can yield variable results owing to differences in motivation amongst individuals or species (Niv et al., 2006). Additionally, methods such as the optomotor assay employ moving stimuli, and so may be affected by an animal's ability to respond to or perceive motion (e.g. Neave, 1984; Westheimer and McKee, 1975), in addition to its acuity. Given the issues associated with each method, best practice may be to use multiple approaches to give a better estimate of acuity in a given species (as in, e.g. Parker et al., 2017).

Fishes in the family Poeciliidae are an ideal system in which to examine relationships between visual signals and visual acuity. They are a model system in the study of mate choice, and use visual signals in both female mate choice and male–male competition. These visual signals can involve colours or patterns (e.g. Basolo and Trainor, 2002; Cummings et al., 2003; Kodric-Brown and Johnson, 2002; Schlüter et al., 1998), structural ornaments (e.g. Basolo, 1990a; Benson and Basolo, 2006; MacLaren et al., 2004; Rosenthal and Evans, 1998; Schlupp et al., 2010) or courtship displays (e.g. Kodric-Brown and Nicoletto, 2001; Kodric-Brown, 1993; Rosenthal and Evans, 1998). Despite the diversity and importance of visual signals in this group, very little is known about visual acuity in the Poeciliidae.

Here, we measured visual acuity in *Xiphophorus helleri* Heckel 1848, the green swordtail, using one anatomical and two behavioural methods. *Xiphophorus helleri* was chosen because the spatial aspects of visual signals play a key role in both female mate choice and male–male competition. In particular, male green swordtails display colourful elongations of their caudal fins, known as swords, and females exhibit preferences for males based on sword length (e.g. Basolo, 1990a,b). Additionally, preferences for larger males are widely present in poeciliids, including in *X. helleri* (e.g. Basolo, 2004; Bischoff et al., 1985; MacLaren, 2017; MacLaren et al., 2011; Rosenthal and Evans, 1998; Ryan and Wagner, 1987). Sword length and body size are also important visual signals in the context of male–male competition. Specifically, males with longer swords experience more aggression from competitors (Franck and Hendricks, 1973; Hemens, 1966) and also have greater competitive success (Benson and Basolo, 2006). To understand how male and female green swordtails perceive traits such as sword length and body size, we used retinal topography to determine RGC peak density and estimate anatomical acuity, and both optomotor assays and conditioned responses to estimate behavioural acuity.

## MATERIALS AND METHODS

### Subjects and housing

All animals were treated in accordance with the ethical guidelines of the University of Exeter (ethics approval eCORN002243). Fish handling and experiments were carried out by E.M.C. (Home Office Personal Licence I56658687) under Home Office Project Licence PF6E68517. All fish used in this experiment were sexually mature descendants of a wild-derived population originally collected in Belize in 2002. Fish were housed in mixed-sex groups of four to seven individuals in 30 litre tanks and fed with a mixture of bloodworm, mysis shrimp and artemia each morning and flake food (ZM Flake, Fish Food and Equipment, Hampshire, UK) each evening. Water temperature was kept between 22 and 24°C and tanks were lit from above with AquaBeam LED lights (Tropical Marine Centre, Herefordshire, UK) on a 12 h:12 h light:dark cycle (although during portions of one Covid-19 lockdown, the light cycle was temporarily shifted to 15 h:9 h light:dark cycle). All fish were tagged with an individually identifiable combination of elastomer tags (Northwest Marine Technology, Anacortes, WA, USA). After the optomotor assay experiment, three males and three females were humanely euthanized in a lethal dose of buffered MS-222, and their heads were fixed in 4% paraformaldehyde in 0.1 mol l^−1^ phosphate buffer for use in preparation of retinal whole-mounts (see below).

### Measuring eye and body size

We used photos of each individual to measure eye and body size. Fish were very briefly removed from their tank, and at least four photos were taken with a size standard present in the frame. For males, we ensured that their sword was fully extended to allow for measurement of sword length. From each photo, we measured standard length (the length from the tip of the snout to the posterior end of the caudal fin, thus excluding the length of the caudal fin; hereafter, body size) and the diameter of the visible portion of the eye (hereafter, eye size) using ImageJ (Schneider et al., 2012). Because multiple images were taken of each individual, we measured eye and body size in each photo and used the average value in analyses. Note that measures of ‘eye size’ were taken from live individuals that were then used further in behavioural trials, meaning we could not dissect out the eye and measure the entire eye or lens directly. By contrast, in a subset of fish that were euthanized and fixed for retinal ganglion cell mapping (see below), lenses were dissected out of the eye cup and their diameter measured using digital callipers, and these measures are referred to as ‘lens size’ throughout.

### Preparation of retinal whole-mounts

Retinal whole-mounts were prepared for three males and three females according to standard protocols (Coimbra et al., 2006; Stone, 1981; Ullmann et al., 2012). During dissection, the orientation of the retina was kept by making a small incision in the dorsal part of the retina. Each retina was bleached overnight at room temperature in a solution of 3% hydrogen peroxide in 0.1 mol l^−1^ phosphate buffer (PBS), rinsed in PBS and whole-mounted, ganglion cell layer facing up, on a gelatinized slide. Each whole-mount was left to dry overnight in formalin vapor to improve fixation and cell differentiation (Coimbra et al., 2006, 2012), stained in 0.1% Cresyl Violet (Coimbra et al., 2006) and coverslipped with Entellan New mounting medium (Proscitech). Because the retinal whole-mount remained attached to the slide during the entire staining process, possible shrinkage during staining was considered negligible and, if present, confined to the retinal margin (Coimbra et al., 2006).

### Stereological analyses and topographic map construction

**Fig. 1.**
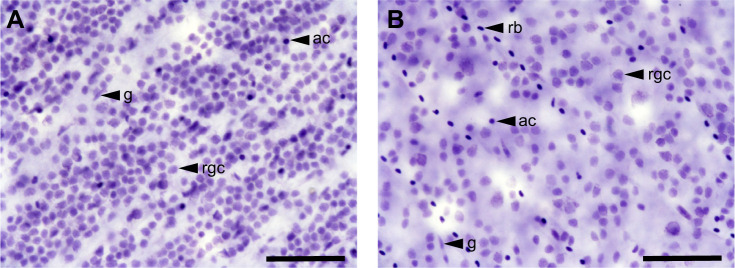
**Light micrographs of the Nissl-stained ganglion cell layer of a representative female swordtail fish.** (A) The peak-density area; (B) a low-density area (ventral). ac, displaced amacrine cells; g, glial cells; rb, red blood cells; rgc, retinal ganglion cells. Scale bar: 50 μm.

Following the protocols described in de Busserolles et al. (2014), topographic distribution of RGCs was assessed using the optical fractionator technique (West et al., 1991) modified by Coimbra et al. (2009, 2012). Briefly, the outline of each retinal whole-mount was digitized using a ×5 objective (numerical aperture 0.16) mounted on a compound microscope (Zeiss Imager.Z2) equipped with a motorized stage (MAC 6000 System, Microbrightfield, USA), a digital colour camera (Microbrightfield) and a computer running StereoInvestigator software (Microbrightfield). RGCs were randomly and systematically counted using a ×63 objective (numerical aperture 1.40). The counting frame and grid size were chosen carefully to maintain the highest level of sampling and achieve an acceptable Schaeffer coefficient of error (CE <0.1; Glaser and Wilson, 1998). A counting frame of 60×60 μm was used for all individuals. The grid size was adjusted between individuals to take into consideration the variation in size between specimens and allow sampling of around 200 sites per retina (Table 1). To estimate the peak density of RGCs, sub-sampling was carried out in the area of highest cell density using the same counting frame size but a smaller grid (i.e. half the size of the original grid in Table 1). RGCs were arranged in a single layer within the ganglion cell layer and were easily identified from displaced amacrine cells and glial cells using cytology criteria alone (Collin and Pettigrew, 1988c; Hughes, 1975) (Fig. 1). Consequently, we are very confident that only RGCs were counted in the analysis.

**Table 1.**
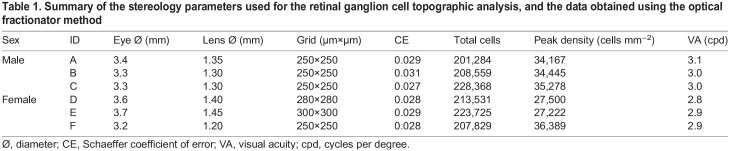
Summary of the stereology parameters used for the retinal ganglion cell topographic analysis, and the data obtained using the optical fractionator method

Topographic maps were constructed in R v.4.1.0 (https://www.r-project.org/) with the results exported from the Stereo Investigator Software according to Garza-Gisholt et al. (2014). The Gaussian kernel smoother from the Spatstat package (Baddeley and Turner, 2005) was used and the sigma value was adjusted to the grid size.

### Estimation of anatomical acuity

The upper limit of acuity in cycles per degree was estimated for each individual using the peak density of ganglion cells (PDG in cells mm^−1^) as described by Collin and Pettigrew (1989). Briefly, the angle subtending 1 mm on the retina (angle α) can be calculated as follows:
(1)

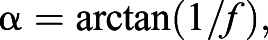
where *f*, the focal length or distance from the centre of the lens to the retina, is 2.55 times the radius of the lens in teleost fishes (Matthiessen's ratio; Matthiessen, 1882). Knowing α, the PDG and the fact that two ganglion cells are needed to distinguish a visual element from its neighbour, the acuity in cpd can be calculated as follows:
(2)

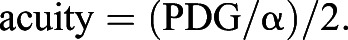


### Behavioural acuity

#### Stimuli

We used data on the maximum and common body size of *X. helleri* (16 cm and 3 cm, respectively) from FishBase.org (Froese and Pauly, 2000), and the relationship between acuity and body size in ray-finned fishes (Caves et al., 2017) to estimate that acuity in *X. helleri* would likely be between approximately 3 and 5 cpd. Therefore, we designed stimuli that, accounting for the viewing distance of 17.5 cm in our experimental setups, were 0.2, 0.4, 0.6, 0.8, 1.0, 2.0, 3.0, 4.0, 5.0 and 6.0 cpd, to both cover and extend beyond the range of predicted acuities. Stimuli were black and white square waves printed using a laser printer (HP Colour LaserJet M553, Hewlett-Packard, USA) on waterproof paper (Premium NeverTear, 120 μm thickness, 160 gsm weight, Xerox, CT, USA). As a control, we used solid grey stimuli. For the optomotor assay, the precise shade of grey was not as important as the absence of any spatial stimuli. Therefore, the optomotor control stimulus was created using cardstock (175 gsm weight, Darice, OH, USA). For the trained two-choice assay, the average brightness of the grey stimulus needed to match the striped stimulus. Therefore, we photographed a printed black and white striped stimulus alongside two grey standards made from Zenith diffuse sintered PTFE sheet (Labsphere, Congleton, UK) with 96.2 and 4.5% reflectance, using a Sony α7 camera fitted with a 28–70 mm lens. We then calibrated the stimulus to the standards to extract the reflectance values of white and black (91 and 6%, respectively) using an image analysis toolbox in ImageJ (Troscianko and Stevens, 2015). To create the grey control stimulus using the mid-point of these values (42.5%), we printed grey squares that incrementally increased in grey value (where a pixel value of 0 is black and 255 is white) using Inkscape. These were photographed to extract reflectance values as described above, and the pixel value that created 42.5% reflectance was identified (pixel value 173).

#### Measuring acuity using an optomotor assay

**Fig. 2.**
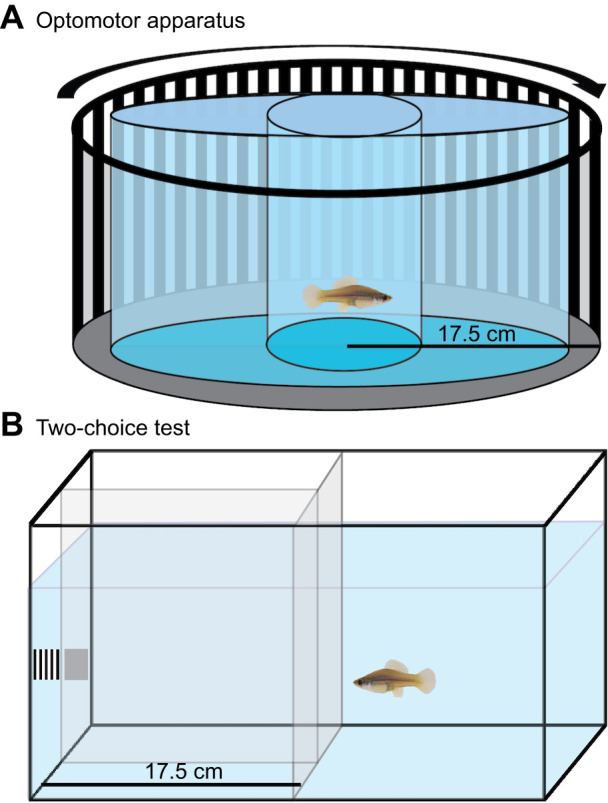
**Schematic of experimental setup.** (A) Optomotor assay; (B) two-choice test. In A, the striped stimulus rotates around the fixed cylindrical testing arena. Variation in viewing distance between the animal and the stimuli was minimized by placing the animal inside a second, smaller cylinder. In B, fish chose to approach either a striped stimulus or a grey stimulus of the same mean luminance, and were rewarded with a bloodworm for correctly approaching the grey (positive) stimulus. Fish initially viewed the stimuli from a given distance by using a central divider and a transparent barrier.

The optomotor apparatus used here is described in detail in Caves et al. (2020). Briefly, during optomotor trials, an individual was placed inside a water-filled 30-cm diameter cylindrical tank made of extruded clear acrylic (which has a low refractive index, minimizing visual distortion when submerged underwater). The experimental tank was placed inside a drum that measured 35 cm in diameter, and which was lined with the square wave stimuli (Fig. 2A). We rotated the drum at a constant speed of 1 rotation min^−1^, an optimal speed for eliciting robust, repeatable responses determined during preliminary experiments. Microstepping allowed the motor to achieve 3200 steps per revolution, maintaining smooth rotation even at low rotation speeds. The optomotor tank was illuminated from directly overhead by a full spectrum arc lamp (Iwasaki EyeColor 6500K, Iwasaki Electroc, Japan) fitted with a 70 W electronic ballast (Venture Lighting Europe, Herefordshire, UK). Light from the arc lamp was filtered through a sheet of 0.25 mm PTFE plastic (Bay Plastics, UK) to provide diffuse, even lighting.

Prior to the beginning of each trial, an individual was placed in the test arena and allowed to acclimate to the tank and the ambient lighting for 10 min, during which time the control stimulus was placed around the tank. At the beginning of the acclimation period, a clear acrylic cylinder 10 cm in diameter was placed around the animal and centred in the testing arena, to minimize the animal's movements towards and away from the stimulus during testing. To begin a trial, a stimulus between 0.4 and 6 cpd was randomly selected and placed around the tank. Starting direction (clockwise or counter-clockwise) was determined randomly using the sample function in R (https://www.r-project.org/). Each stimulus was rotated for 45 s in the starting direction, and then after a 5 s break, rotated for 45 s in the opposite direction. That pattern was then repeated, so in total, each fish saw each stimulus for two clockwise and two counter-clockwise rotations. Fish were then given 2 min before a second randomly selected stimulus was presented, and so on.

Fish were tested on all stimuli during a single trial that lasted approximately 1 h. To end the trial, fish were shown the 0.2 cpd stimulus, the stimulus with the thickest stripes, which elicited robust responses during preliminary trials. This stimulus was presented last to ensure that fish had remained motivated or capable of performing optomotor responses throughout the trial; all fish exhibited positive responses to the 0.2 cpd stimulus. The grey control was used once during each trial. Temperature was noted at both the start and end of a trial, and remained between 22 and 24°C throughout.

All trials were video recorded from above using a GoPro camera (Hero 3+, GoPro, CA, USA). Videos were annotated by two viewers who were blind to the identity and direction of rotation of the stimulus. First, viewers noted whether fish exhibited stress behaviour during any one of the four rotations, defined as quick, random darting motion around the tank; any rotation in which stress behaviour was observed (which occurred in 94 out of 1895 total rotations) was not used in analyses. Viewers also noted whether eye tracking was visible during the rotation, the direction of the fish's rotation, and whether it rotated 0 to one-quarter turn, one-quarter to one-half turn, one-half to one full turn, or more than one full turn. Between the two viewers, there was 87.9% agreement in annotations, so the annotations from the viewer with more experience with optomotor assays were used for analysis.

Based on preliminary trials with the 0.2 cpd stimulus and rotation at 1 rotation min^−1^, a positive response was determined to occur if either eye tracking or at least one-half turn in the direction of stimulus rotation occurred in at least three out of the four trials. The acuity limit was taken to be the finest stimulus to which a fish exhibited a positive response. In total, we collected optomotor data from 28 fish (13 males and 15 females). Of these, two females exhibited positive responses to the control stimulus and so were excluded from data analysis. Additionally, one female was identified as an outlier using Cook's distance (Cook, 1977), and so was not included in analyses, although including her in the analyses did not change the conclusions in any instance. Thus, final sample sizes for analysis of optomotor acuity were 13 males and 12 females.

All analyses were run in R version 4.0.3 (https://www.r-project.org/). To statistically analyse differences in optomotor acuity between males and females, we used a non-parametric Kruskal–Wallis rank sum test, because the assumptions of a parametric test were not met. Specifically, acuity in males was not normally distributed (Shapiro–Wilk normality test, *W*=0.81, *P*=0.01), and variance in acuity in males and females was not equal (*F*-test for homogeneity in variances, *F*_12,12_=8.01, *P*=0.001).

We built linear models using the lm function in R to examine relationships between eye size, body size and optomotor acuity. First, we asked whether the relationship between eye size and body size differed between males and females, by fitting models in which eye size was the response variable and both body size and sex were fixed effects. Second, we examined whether the relationship between acuity and either eye or body size differed between males and females by fitting models in which acuity was the response variable and both sex and a measure of size (either eye size or body size) were fixed effects.

For each analysis, we first fitted a full model including both fixed effects and their interaction. After fitting a full model, we used the ‘drop1’ function in the lme4 package (Bates et al., 2015) which uses an *F*-test to determine the significance of each fixed effect by comparing the full model to a model without each fixed effect in turn. To avoid problems with stepwise model reduction (Mundry and Nunn, 2009), we removed non-significant interactions to test the main effects, but did not remove non-significant main effects. To confirm that the assumption of normality in the residuals was met, we used diagnostic plots and performed Shapiro–Wilk normality tests on model residuals.

#### Measuring acuity using a conditioned discrimination assay

As a second way to behaviourally measure acuity, we used a conditioned discrimination assay in which fish were trained with food rewards to discriminate a neutral grey stimulus from a striped stimulus. Throughout training and testing, fish were moved to 15-litre tanks in which they were physically, but not visually or chemically, isolated from other fish. Modified Y-mazes were built in each tank to control the distance at which fish first viewed the striped stimuli (Fig. 2B). The Y-mazes comprised a central divider that created two compartments (left and right) which the fish observed from behind a clear acrylic barrier. The central divider was 17.5 cm in length, so when the fish viewed the left and right compartments from behind the acrylic barrier, the viewing distance was approximately 17.5 cm, the same viewing distance as in the optomotor trials.

At the beginning of each trial, opaque dividers were placed in between neighbouring tanks to visually isolate fish from one another. A GoPro camera (Hero3+) was placed at the back of each tank to record the trials. Because acuity can vary with the brightness of ambient light (e.g. Abdeljalil et al., 2005; Caves et al., 2016), during trials, each tank was lit from above with an LED light panel (Hersmay CHD-160A) fitted with a diffuser, to achieve the same overall brightness (measured in lux) as in the optomotor assay. Fish were given 10 min to acclimate to the lighting conditions and the presence of the camera before trials were run.

Prior to each trial, the sample function in R was used to randomly assign the grey and grating stimuli to the left or right compartments. To start a trial, the clear barrier was slid into position to keep the fish at least 17.5 cm from the stimuli, and the stimuli were displayed in the left and right compartments, at which point the fish were allowed approximately 20 s to view the stimuli before the barrier was raised. The fish was given up to 3 min to make a choice by approaching one stimulus or the other. The choice was taken as the first compartment entered by a fish after raising the barrier. If the fish correctly selected the grey (positive) stimulus, it was rewarded with a bloodworm.

Initially, to condition fish to associate the grey stimulus with a reward, we displayed only grey stimuli until the fish would reliably approach the stimuli after the barrier was lifted. We then trained the fish to discriminate the grey stimulus from the grating stimulus by offering them a choice between the grating and the grey stimuli, but rewarding only visits to the grey stimulus. The fish were trained in this manner until they chose the grey stimulus in a minimum of six out of seven consecutive trials (a rate of 85% correct choices). Owing to ethical regulations regarding the physical isolation of fish, training and data collection had to be carried out during a 3-week period. Therefore, any fish that had not achieved the pass criterion in training trials at the end of 2 weeks were not advanced to data collection, to allow sufficient time to collect data. In total, we were able to train and collect data on four males and four females; three males and two females that began training did not pass to data collection.

During data collection, fish first received five ‘refresher’ trials with the 0.2 cpd stimulus. This was followed by 5 to 10 trials of another stimulus, with 1 min between successive presentations of a given stimulus. Following a 2 min break, fish were given 5 to 10 trials of another stimulus, and so on. To confirm that fish had maintained motivation throughout, they were offered a bloodworm in the absence of any stimuli at the end of each trial day; all fish had maintained motivation during the trials. Stimuli were presented in random order. We also randomized which side of the tank (left or right) the positive and negative stimuli were presented on, resulting in a mean±s.d. of 48.7±0.03% of trials in which the positive stimulus was presented on the left-hand side of the tank (range: 44.4–53.8%).

We aimed for each individual to receive 15–20 trials with each grating width, but ethical restrictions on the length of time for which fish could be isolated meant that some fish did not achieve the target number of trials (median: 15 trials per stimulus per individual; range: 9–21). A fish was deemed to have been able to discriminate the striped stimulus from the grey stimulus significantly better than chance if their success rate was 73% or higher (chosen as the threshold for significant deviation from chance in a two-alternative forced choice test, based on a binomial test, *n*=15 trials, *P*<0.05).

## RESULTS

### RGC topographic distribution and anatomical acuity

**Fig. 3.**
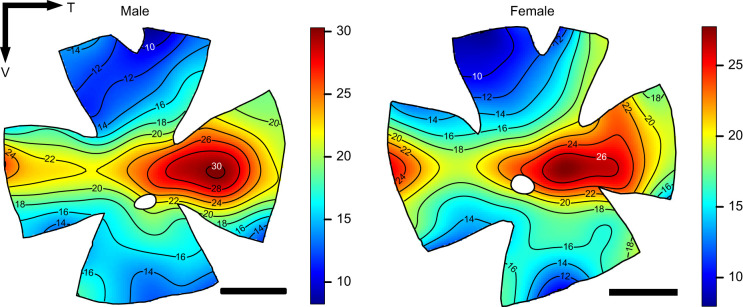
**Topographic distribution of RGCs in the retina of the green swordtail *Xiphophorus helleri*, for one representative male (left) and one representative female (right).** The black lines represent iso-density contours and values are expressed in densities ×10^3^ cells mm^−2^. The white spot in the middle of each retina is the optic nerve. The black arrow indicates the orientation of the retinas. T, temporal; V, ventral. Scale bars: 1 mm.

The topographic distribution of RGCs was investigated in three males and three females. Overall, intraspecific variability in RGC distribution and density was very low and no obvious differences between sexes were observed. A representative RGC topographic map for one male and one female is shown in Fig. 3, and maps for all individuals are provided in Fig. S1. All individuals presented the same topography pattern characterised by two areas of high cell densities, an elongated area in the centro-ventral part of the retina where the peak density of RGC lies and a smaller area in the nasal part, as well as a weak horizontal streak.

The total number of RGC was very similar between individuals with a mean total number of 213,883 (Table 1). Peak cell density varied slightly between individuals from 27,222 to 36,389 cells mm^−2^. This variation can be explained by differences in individual size and therefore eye size, with bigger fish having a bigger retinal area and therefore lower cell densities (Table 1). Based on the peak cell density located in the dorso-temporal area and the lens diameter of each individual, visual acuity was estimated to be approximately 3 cpd for all individuals (mean±s.d.: 3.0±0.06 cpd in males, 2.9±0.06 cpd in females; Table 1).

### Optomotor acuity, eye size and body size

**Fig. 4.**
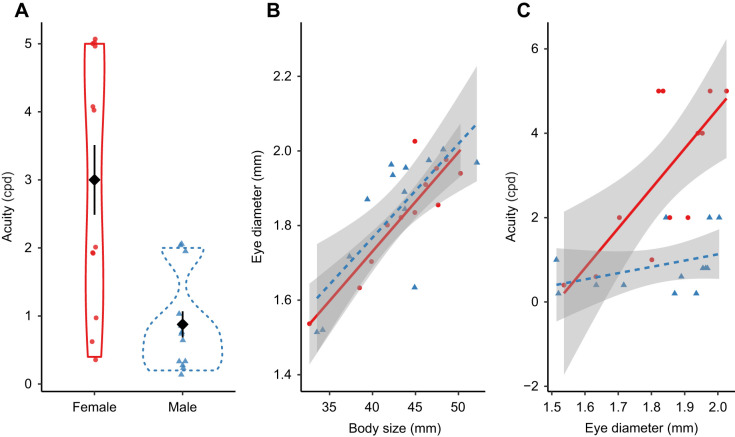
**Optomotor acuity, eye size and body size in male (blue dashed line and triangles, *n*=13) and female (red solid line and circles, *n*=12) green swordtails.** (A) Mean optomotor acuity in females is significantly higher than in males (non-parametric Kruskal–Wallis rank sum test, χ^2^_1_=8.67, *P*=0.003). Diamonds represent means and lines represent standard error. (B) The relationship between eye diameter and body size does not differ in males and females, although (C) for a given eye size, females have higher acuity. In B and C, the grey shaded regions show 95% confidence intervals.

Acuity estimates obtained from an optomotor assay were higher in females (*n*=12; mean±s.d.: 3.00±1.85 cpd; median: 3.0 cpd; range: 0.4–5 cpd) than in males (*n*=13; 0.88±0.69 cpd; median: 0.8 cpd; range: 0.2–2 cpd), and this difference was significant (χ^2^_1_=8.67, *P*=0.003) (Fig. 4A).

Males and females did not differ significantly, however, in either eye size (females: 1.83±0.15 mm, males: 1.83±0.18 mm; χ^2^_1_=0.11, *P*=0.75) or body size (females: 43.8±4.98 mm, males: 42.5±5.31 mm; χ^2^_1_=0.67, *P*=0.41). Additionally, the relationship between eye and body size did not differ between males and females (Fig. 4B, Table 2). In a model that included eye size as the response variable, and body size, sex and their interaction as fixed effects, the interaction term was not significant (*F*_1_=0.03, *P*=0.86). After dropping the non-significant interaction term, we found that in an additive model, body size, but not sex, was a significant predictor of eye size (body size: *F*_1_=45.44, *P*<0.0001; sex: *F*_1_=0.71, *P*=0.40).

**Table 2.**
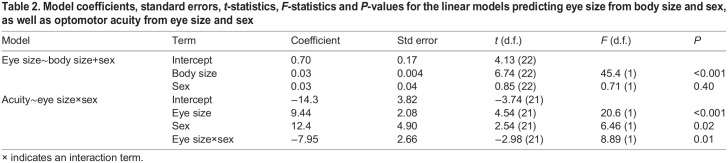
Model coefficients, standard errors, *t*-statistics, *F*-statistics and *P*-values for the linear models predicting eye size from body size and sex, as well as optomotor acuity from eye size and sex

The relationship between acuity and eye size, however, did differ between males and females (Fig. 4C). In a model that included acuity as the response and eye size, sex and their interaction as predictors (Table 2), the interaction term and both main effects were significant (eye size: *F*_1_=20.59, *P*<0.001; sex: *F*_1_=6.46, *P*=0.02; eye size×sex interaction: *F*_1_=8.89, *P*=0.007). Together, these results show that mean acuity in females is higher than in males, and that females have higher acuity than do males for a given eye size. However, higher acuity in females is not attributable to females having relatively larger eyes for a given body size than do males.

### Conditioned acuity

**Fig. 5.**
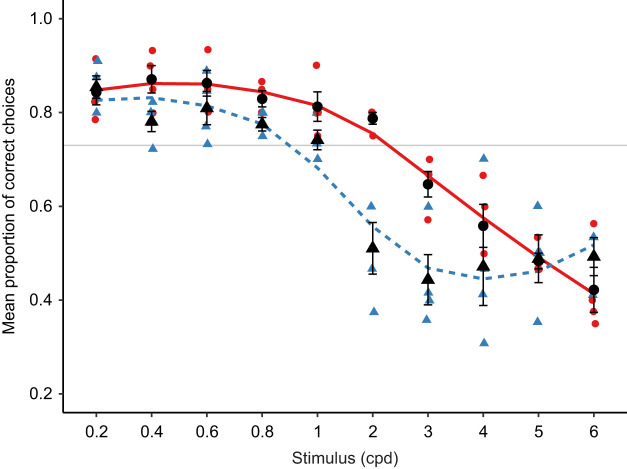
**Acuity estimates from a conditioned discrimination assay in green swordtail females (red solid line, circles, *n*=4) and males (blue dashed line, triangles, *n*=4).** Black symbols show means, error bars show standard errors. Trendlines for males and females were fitted using LOESS smoothing. The horizontal grey line is at 0.73, the threshold criterion above which fish performed significantly better than chance at discriminating between solid grey and grating stimuli.

Using an optomotor assay, we found evidence for higher acuity in females than in males. To examine further whether this was a true sexual dimorphism in acuity, or whether it had arisen as a result of behavioural differences between the sexes during the optomotor assay, we measured acuity using a conditioned discrimination assay. Four males and four females were tested on their ability to discriminate grating stimuli from a grey stimulus of the same mean luminance. Within each sex, the mean proportion of correct choices (based on which stimulus they approached first in a Y-maze setup) is shown in Fig. 5.

In males, the highest proportion of correct choices (0.85±0.05) occurred in response to the stimulus with the lowest spatial frequency (0.2 cpd), whereas in females, the highest proportion of correct choices (0.87±0.06) occurred at 0.4 cpd. As a group, males maintained a mean proportion of correct choices above the threshold criterion of 0.73 (see Materials and Methods) until between 1 and 2 cpd (at 1 cpd: 0.74±0.04; at 2 cpd: 0.51±0.11). Three out of four males performed above the threshold criterion at 1 cpd, whereas all four performed below threshold at 2 cpd. On average, females maintained performance above the threshold criterion until between 2 and 3 cpd (at 2 cpd: 0.79±0.03; at 3 cpd: 0.65±0.05). All four females performed above the threshold criterion at 2 cpd, but all performed below threshold at 3 cpd. Thus, acuity limit estimates using conditioned discrimination assay were between 1 and 2 cpd in males, and between 2 and 3 cpd in females.

## DISCUSSION

The acuity estimates presented here for the green swordtail *X. helleri* are the first for any fish in the genus *Xiphophorus*, a genus in which ornaments such as sword length serve as important mate choice and agonistic signals (e.g. Basolo, 1990a,b). They are also some of the first measures of acuity in the family Poeciliidae, a model family in the study of how sexual and natural selection influence visual signal evolution. Our primary finding is that estimates from one anatomical and two behavioural methods suggest that acuity in swordtails is between 1 and 3 cpd. These estimates align well with predicted acuity based solely on body size in green swordtails (see Caves et al., 2017) and are also similar to acuity estimates from other small, freshwater fish, for example, Japanese rice fish (∼1 cpd; Carvalho et al., 2002), goldfish (*Carassius auratus*, ∼1 cpd; Hodos and Yolen, 1976), zebrafish (*Danio rerio*, ∼2 cpd; Pita et al., 2015) and cutlips minnow (*Exoglossum maxilingua*, ∼3 cpd; Collin and Ali, 1994). Second, while anatomical acuity was found to be identical in both males and females (∼3 cpd), females had behavioural acuity estimates that closely aligned with the anatomical predictions (3 cpd), while male behavioural estimates were lower (1–2 cpd), suggesting that behavioural acuity may be sexually dimorphic in this species. Lastly, RGC topography was characterized by areas of high cell densities located centro-temporally and nasally, as well as a weak horizontal streak. The retinal maps presented here are the first such topographic maps for the family Poeciliidae.

### Retinal topography

RGC topography often relates to an animal's ecology through the ‘terrain theory’ (Hughes, 1977), which suggests that the organization of RGCs reflects the physical structure of an animal's environment. For example, fish species that live in spatially complex habitats such as coral reefs often have well-developed ‘areas’ of acute vision (Collin and Pettigrew, 1988a). Conversely, fish living in open environments with an uninterrupted view of the horizon, such as pelagic or bottom-dwelling species, often possess a horizontal ‘streak’ that allows them to view a broad area of the horizon with enhanced acuity, without the need for distinctive head and eye movements (Collin and Pettigrew, 1988b; Collin and Shand, 2003).

However, aside from the environment, several other ecological factors, such as feeding mode (de Busserolles et al., 2014, 2021; Luehrmann et al., 2020), predator detection (Collin and Pettigrew, 1988a), competition (Stieb et al., 2019) and signalling traits (Parker et al., 2017), may also contribute to the evolution of specific retinal topography patterns. In the Ambon damselfish, *Pomacentrus amboinensis*, for example, the higher density of RGCs in the dorso-temporal part of the retina that extends centrally (Parker et al., 2017) may enhance their ability to discriminate the species-specific fine facial markings of their conspecifics (Siebeck, 2004; Siebeck et al., 2010). In the anemonefish *Amphiprion akindynos*, the horizontal streak may allow them to detect intruders from the safety of their anemone, while the peak density of RGCs located in the central part of their retina may be useful in assessing conspecific body size during side-by-side swimming competitive encounters (Stieb et al., 2019).

Similarly, the centro-temporal elongated area and the weak horizontal streak found in *X. helleri* may enhance their ability to detect and discriminate sword length and/or body size, both of which are important visual signals that are horizontally oriented in the fish's field of view during displays (e.g. Basolo, 1990a; Hemens, 1966; Rosenthal et al., 1996; Ryan and Causey, 1989). During courtship, for example, a male *X. helleri* will often orient himself transversely in front of a female and then wrap his body and sword in a U-shape around the head of the female, presenting his entire body and sword laterally in the female's field of view (Hemens, 1966). During male–male contests, swordtail males will often approach one another and display in a lateral orientation (as in *X. nigrensis*; Moretz, 2003), and the size of the opponents' bodies (as in *X. nigrensis*; Moretz, 2003) and swords (as in *X. helleri*; Benson and Basolo, 2006) play a role in determining which individual initiates and wins a contest. However, given the lack of data on retinal topography in the genus *Xiphophorus*, or poeciliids more broadly, we cannot be certain whether the topography pattern observed in *X. helleri* is a widely shared trait, or a specific adaptation for signal detection and assessment. Further comparative work on related species with different signal forms (such as spots in the guppy *Poecilia reticulata* or vertical bars in the humpbacked limia, *Limia nigrofasciata*) will lend further insight.

### Anatomical versus behavioural acuity

Behaviourally, both an optomotor and a conditioned discrimination assay indicated that acuity is approximately 3 cpd in females, but approximately 1 cpd in males. There is inconsistency in the literature regarding whether acuity estimates from RGC density and behavioural assays align closely, although increasingly, data on acuity measured using both RGC density and behavioural methods are becoming available for species with camera-type eyes. Outside of fish, several studies have found close alignment between RGCs and behavioural estimates. For example, close agreement was found between RGC acuity and acuity using a conditioned discrimination assay in the fat-tailed dunnart (*Sminthopsis crassicaudatua*, RGC: 2.3 cpd, behaviour: 2.36 cpd; Arrese et al., 1999), the northern quoll (*Dasyurus quoll*, RGC: 2.6 cpd, behaviour: 2.8 cpd; Harman et al., 1986) and the northern leopard frog (*Rana pipiens*, RGC 2.82 cpd, behaviour: 2.80 cpd; Aho, 1997). Similarly, some studies have found close agreement between RGC and optomotor acuities, as in Anna's hummingbird (*Calypte anna*, RGC: 5.3 cpd, optomotor: 6 cpd; Goller et al., 2019) and the common chameleon (*Chamaeleo chameleon*, RGC: 7.4 cpd, optomotor: 9 cpd; Lev-Ari et al., 2017).

In fish, however, only a small number of studies have compared RGC acuity with acuity measured using a conditioned choice assay (data comparing RGC acuity with optomotor acuity in a single study are lacking), and in general they have found estimates from RGC density to be higher than behavioural estimates. For example, in the Ambon damselfish, acuity calculated using RGCs was 4.1 cpd, but only 1.36 cpd when measured using a conditioned discrimination assay (Parker et al., 2017). Similar differences were found in the brown dottyback (*Pseudochromis fuscus*, RGCs: 3.6 cpd, behaviour: 1.69 cpd; Parker et al., 2017) and the triggerfish *Rhinecanthus aculeatus* (RGCs: 3.41 cpd, behaviour: 1.75 cpd; Champ et al., 2014). In our study, however, RGC acuity was nearly identical to that derived from both behavioural methods, but only in females. In males, acuity estimated from RGCs was approximately three times higher than behavioural estimates.

There are several reasons why anatomical and behavioural methods may yield different estimates. First, not all ganglion cells contribute to acuity, so acuity estimates based on counts of all ganglion cells represent a theoretical upper limit on acuity, and behavioural acuity may be lower. Second, RGC density counts cannot take into account any processing of spatial information that occurs beyond the retina, which could affect an animal's spatial perception and subsequent behaviour. Third, the experimental parameters used in behavioural assays, as well as the criteria for what constitutes a positive response, can affect acuity estimates. For example, the brightness of the ambient illumination and acclimation to experimental light conditions can affect acuity estimates (e.g. Abdeljalil et al., 2005; Caves et al., 2016; Thaung et al., 2002). We attempted to control for these factors in both of our behavioural assays by ensuring the brightness of the lighting conditions, as well as acclimation time, were the same in the optomotor and conditioned discrimination assays. In the conditioned assay, the choice of which cut-off threshold criterion to use can affect acuity estimates. Discrimination thresholds in similar assays have been commonly set at between 70 and 75% (e.g. Hodos and Yolen, 1976; Lind and Kelber, 2011; Parker et al., 2017; Potier et al., 2016; Temple et al., 2013), or as low as 65% (Champ et al., 2014). However, applying a 65% criterion to our data would not change our acuity estimates.

Although behavioural acuity estimates are often lower than anatomical estimates, several individuals in this study exhibited higher optomotor than anatomical acuity. One potential explanation for this is that there may have been slight positional differences between individuals during optomotor assays, which we could not avoid without physically restraining the fish's movements. Although we attempted to minimize variation in viewing distance, some fish may have moved slightly closer to the stimulus than others, resulting in higher apparent acuity. Additionally, optomotor assays inherently may confound an animal's ability to resolve detail with its ability to respond to motion, potentially making some rotating stimuli more resolvable than they would be when static (Nakayama, 1985; Douglas and Hawryshyn, 1990). We did, however, attempt to minimize these confounding effects by rotating the stimuli at the very low rate of 1 rotation min^−1^, the slowest possible rotation speed that elicited a robust response during preliminary trials. Overall, however, our results suggest that with sufficient sample sizes, mean acuity obtained using an optomotor assay can align closely with acuity measured using other methods.

### Sexual dimorphism in acuity

Regardless of why anatomical and behavioural estimates may not align, it is interesting that in this study the misalignment occurred only in males, and not in females. Eye and body size are known drivers of variation in acuity across species (Caves et al., 2018), and in some cases sexual dimorphism in eye or body size may explain sexual dimorphism in acuity. For example, one recent study of guppies (*Poecilia reticulata*) found higher optomotor acuity in females than in males, although this dimorphism was attributable to females having overall larger body sizes (Corral-López et al., 2017). In this study, however, we observed no significant differences between males and females in either eye or body size; rather, for a given eye size, females exhibited higher acuity than males.

Sexual dimorphism in visual acuity is known primarily from insects, in which the morphology of the compound eye, and thus visual acuity, can differ dramatically between males and females. For example, in the bilobed eye of male march flies and mayflies, the upper lobe has been flattened to increase retinal sampling density within a narrow, upward field of view (Zeil, 1983a), which is useful when spotting small, fast-moving mates against the sky (Zeil, 1983b). In male hoverflies in the genus *Volucella*, males have frontally oriented areas of heightened acuity that are more acute than equivalent zones in females (Warrant, 2001). In the same vein, even though total ganglion cell density and distribution was similar between sexes, female swordtails may have a higher proportion of ganglion cell subtypes involved in acuity tasks than males. This would explain why the sexual dimorphism was only detected behaviourally in our study.

Another possibility, given that retinal topography in males and females was essentially identical, is that the sexual dimorphism observed here occurs as a result of behavioural or attentional differences between males and females during experimental assays. Behavioural acuity in males might approach anatomical acuity if a different method was used to measure acuity, particularly if a method made use of more naturally relevant behaviours than grating acuity. There could also be differences in post-retinal processing between males and females. For example, goldfish exhibit better binocular acuity than monocular acuity, implying that there may be some central contribution to acuity processing (Penzlin and Stubbe, 1977). However, how higher-order processing of spatial information may explain differences between anatomical and behavioural acuity, or differences in acuity between sexes, is not well understood. Overall, two distinct behavioural tasks that measured grating acuity in different ways (the innate optomotor response versus a conditioned discrimination task) yielded the same difference in acuity between males and females (but not always the same acuity estimates in each individual; see Table S1), lending support to the conclusion that there is some sexual dimorphism in acuity in this species.

### Implications for signalling

**Fig. 6.**
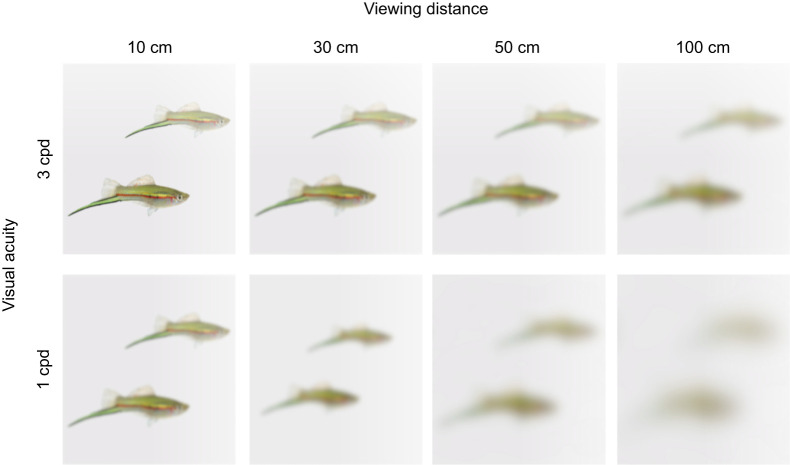
**Two male green swordtails *Xiphophorus helleri*, which differ in sword length and body size, as the spatial aspects of this scene might appear at four different viewing distances to viewers with acuity of 3** **cpd (as in females; top row) or 1** **cpd (as in males; bottom row).** These images were created using the R package AcuityView (Caves and Johnsen, 2017), which uses Fourier methods to delete spatial frequencies from an image that are below the acuity of a given viewer from a given distance. In AcuityView, the image was assumed to be 12 cm across, resulting in the two male swordtails having swords that differ in length by 5 mm, as in the example in the Discussion (Implications for signalling).

The acuity estimates reported here have implications for how male and female swordtails might resolve one another's signals during mate choice and agonistic encounters. Because acuity is distance dependent (Fig. 6), smaller differences are resolvable from closer viewing distances. Thus, acuity can play an important role in determining a signal's active space, or the area of which a signal can propagate and be detected by a receiver (Gerhardt, 2009). For example, for a viewer with acuity of 3 cpd, such as that found in females both anatomically and behaviourally, a 5 mm difference in sword length between two males would become unresolvable at a distance of 86 cm. For a viewer with acuity of only 1 cpd, such as that found behaviourally in males, that same difference would become unresolvable at only 29 cm (for calculation methods, see Coimbra et al., 2015).

Although detailed information on courtship distances in *X. helleri* is lacking, Hemens (1966) reported that the majority of active courtship involved males either resting at approximately 10 cm distance from the female or actively courting her at a distance of only 2–3 cm. However, before active courtship begins, females may benefit from increased acuity, and the ability to detect males and discriminate differences between potential mates from greater distances, for several reasons. First, detecting a male from a greater distance may help females avoid costly aggression or harassment (e.g. Magurran and Seghers, 1994a). Second, females may benefit from detecting an undesirable mate and thus avoid him approaching too closely, to minimize the chances of forced copulation, a common strategy in many fish (Ryan and Causey, 1989), including in the genus *Xiphophorus* (e.g. Morris et al., 2008; Ryan and Causey, 1989; Zimmerer and Kallman, 1989). Lastly, close display behaviour by males, which is a highly conspicuous activity, may attract the attention of predators and therefore increase the risk of predation to both parties (e.g. Magurran and Seghers, 1994b); if a female is able to determine from a greater distance whether she is interested in a given male, she could minimize this risk. Males, in contrast, may not benefit from higher acuity. Because mating in *X. helleri* involves internal fertilization, mating requires physical contact between male and female, so males will nearly always benefit from closely approaching females. Additionally, in many poeciliid fishes, males exhibit preferences for larger females (e.g. Basolo, 2004; Houde, 1987), which are also more fecund, although Tudor and Morris (2009) suggest that male swordtails determine which females are more fecund using non-visual information, such as olfactory cues. Male–male competition also appears to mostly occur at close distances, and thus does not require high acuity. In staged encounters between male *X. cortezi*, Moretz (2003) found that competitive bouts began with close approaches, and the majority of fights escalated to biting and continued displaying, all of which occurred at close range.

Of note, however, is that the behavioural acuity measurements reported here were collected under full-spectrum lighting in the shallow, clear, well-lit environment of our aquarium system, and from controlled, standardized viewing distances. Various aspects of a species' environment can of course affect its visual capability. Of particular relevance to many fish is turbidity; in turbid conditions, light scatters from suspended particles, attenuating high spatial frequencies and affecting the ability to resolve small details (Gazey, 1970; Wells, 1969). Additionally, acuity varies with changes in light level, as occurs in neighbouring shaded versus sunny patches, which can impact courtship display distance (as in guppies; Long and Rosenqvist, 1998). Therefore, acuity in natural environments may be lower than that measured here, underscoring the importance of considering the environment in which signalling occurs before extrapolating from the measures generated in the laboratory to natural signalling behaviours.

Overall, this is one of the first studies of visual acuity in the family Poeciliidae, and lends insight into how this group may perceive visual signals during interactions with important fitness consequences, such as mate choice and aggression. This is also one of the first studies in fish to measure acuity in the same species using three different methods, and lends insight into how estimates generated using different experimental paradigms may compare with one another. We also found evidence that behavioural acuity in this group may be lower in males than in females. If the sexually dimorphic acuity in swordtails observed in our behavioural assays extends to ecologically relevant signalling interactions, even the relatively small difference between visual acuity of 1 and 3 cpd can have implications for the information male and female viewers can potentially extract from visual scenes.

## Supplementary Material

Supplementary information
