# Supplementary material for: Sex differences in behavioural and anatomical estimates of visual acuity in the green swordtail, Xiphophorus helleri
Source: J Exp Biol. 2021 Dec 17;224(24):jeb243420. doi: 10.1242/jeb.243420 (PMC8729911; doi:10.1242/jeb.243420)
Supplement: Supplementary information [file jexbio-224-243420-s1.pdf]

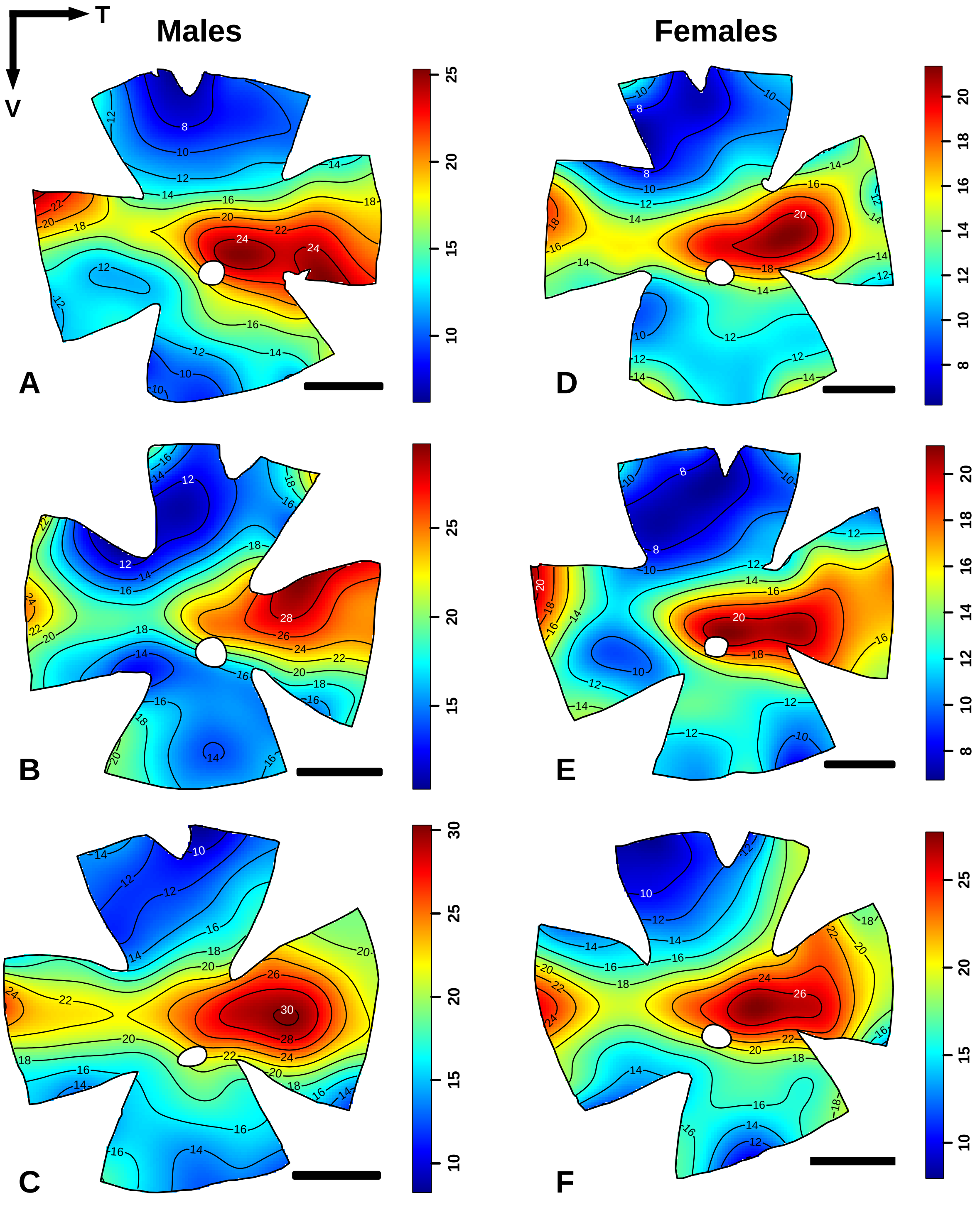

Fig. S1. Topographic distribution of RGCs in the retina of the green swordtail, *Xiphorphorus helleri*, for each of the three males (A-C) and three females (D-F) examined in this study. The black lines represent iso-density contours and values are expressed in densities $\times 10^3$ cells mm^{-2}. The white spot in the middle of each retina is the optic nerve. The black arrow indicates the orientation of the retinas. T, temporal; V, ventral. Scale bars = 1 mm.

Journal of Experimental Biology • Supplementary information

Table S1. Comparison of behavioural acuity in individuals measured using both an optomotor assay and the trained grating choice method. Note that in some cases elastomer tags changed during the experiment (were added or lost); to ensure that these data are comparable with those in the supplemental R code analysis file, alternate Fish ID's are also given here.

Fish ID	Sex	Optomotor Acuity (cpd)	Grating choice acuity (cpd)	Alternate Fish ID
**BlueBlue**	Female	5	2	
**BluePink**	Female	2	2	
**GreenBlue**	Female	5	2	GreenGreen
**RedRed**	Female	1	2	
**BlueBlueBlue**	Male	0.8	1	X1C Male 1
**BlueGreen**	Male	1	1	X3C Male 2
**Green**	Male	0.6	1	GreenGreen
**RedOrange**	Male	2	0.8	
